# The Utility of Cannabis-Based Medicine in Chronic Pain Management: A Case Report

**DOI:** 10.7759/cureus.31555

**Published:** 2022-11-15

**Authors:** Gretchen E Maurer, Nicholas S Imperato, Cameron M Juybari, Hope Kincaid, Andrew Koons

**Affiliations:** 1 Family Medicine, Lehigh Valley Health Network/University of South Florida (USF) Morsani College of Medicine, Allentown, USA; 2 Emergency and Hospital Medicine, Lehigh Valley Health Network/University of South Florida (USF) Morsani College of Medicine, Allentown, USA; 3 Network Office of Research and Innovation, Lehigh Valley Health Network/University of South Florida (USF) Morsani College of Medicine, Allentown, USA

**Keywords:** prescribed cannabis, opioid alternative, opioid use, cannabis medicine, chronic pain

## Abstract

Chronic pain is a common diagnosis that patients may face, resulting in increased morbidity and mortality and affecting the overall quality of life. In addition to established multidisciplinary pain management, medical cannabis may offer an approach to improving pain outcomes and functionality. This case involves a 72-year-old female patient, with chronic neck, lower back, and diffuse arthritic pain due to comorbid osteoarthritis (OA), scleroderma, and scoliosis. Medical cannabis therapy was certified based on the goals of improving pain control and simultaneously reducing the patient’s chronic opioid medication dose. Using potential opioid alternatives, such as medical cannabis, may prove beneficial to clinicians looking to improve pain management and reduce opioid therapy in patients.

## Introduction

The US Centers for Disease Control and Prevention (CDC) has stated that roughly 20% of Americans suffer from chronic pain and it is one of the most frequent reasons adults seek medical care [1,2]. Chronic pain is defined as pain with a duration of at least three months that is persistent even after the resolution of the initial cause [3]. Prevalence estimates for chronic pain vary; a survey-based study reports that as many as 8% of the US population had chronic pain [4]. A published manuscript on pain management and opioid regulation discussed the limitations of opioid alternatives [3]. Therefore, analgesia modalities were trailed in this publication before patients were deemed unable to find effective treatments for chronic pain [3]. For many years, opioid therapy was used to treat various diagnoses that led to pain [5]. This eventually led to the overprescription of opioids and the trickling effect of the opioid epidemic [5]. In recent years, therapeutic approaches to chronic non-cancer pain shifted away from opioid therapy as first line due to increased risks of side effects, morbidity, and mortality [6]. The new therapeutic focus is on non-opioid pharmaceuticals such as nonsteroidal anti-inflammatory drugs (NSAIDs), gabapentinoids, tricyclic antidepressants, and serotonin/norepinephrine reuptake inhibitors [6].

Cannabinoid use as recommended by a doctor in the treatment of a medical condition has been utilized as an approach to effectively manage chronic pain [6]. Cannabinoid therapy provides an analgesic effect through the activation of cannabinoid receptors 1 and 2 (CB1/CB2) and is ubiquitous in the central and peripheral nervous system [7]. This in return impacts the inflammatory processes respectively within the endocannabinoid system (ECS) [7]. While CB1 is abundant, other factors are present in pathways that impact pain perception; for instance, cyclooxygenase-1 (COX-1) and cyclooxygenase-2 (COX-2) are enzymes that promote inflammation [8]. Furthermore, cannabidiol (CBD) inhibits the activation of COX-1 and COX-2, ultimately leading to less inflammation [8]. While details of pain pathways are outside the scope of this manuscript, it deserves mentioning that other elements, including but not limited to nuclear factor kappa B (NFkB) and prostaglandin E (PGE), have links to pain perception [9,10]. CB1 receptors are the most prevalent G protein-coupled receptors within the central nervous system involved in the mitigation of pain and the modulation of these receptors and may prove to be a beneficial target of cannabis [7].

The two main cannabinoids include tetrahydrocannabinol (THC) and cannabidiol (CBD) and work through interaction with the endocannabinoid system to inhibit inflammation and the sensation of pain [11]. THC interacts with both CB1 and CB2 receptors as a partial agonist (higher binding affinity, CB1>CB2) in the central and peripheral nervous system with effects on analgesia and inflammation by reducing the inflammatory cascade. On the other hand, CBD can mitigate the effects of THC through negative allosteric modulation at CB1 or CB2. CBD has been found to interact with multiple other targets including 5-hydroxytryptamine 1A (5-HT1A) receptors in addition to multiple ion channels (transient receptor potential vanilloid 1 {TRPV1}, transient receptor potential ankyrin 1 {TRPA1}, transient receptor potential melastatin 8 {TPRM8}, and glycine receptor {GlyR}), peroxisome proliferator-activated receptors (PPARs), enzyme inhibition at fatty acid amide hydrolase (FAAH) (enzyme responsible for breaking down endocannabinoids), and likely additional unknown pathways [11]. This case presents a 72-year-old female who had previously failed to achieve adequate pain control and desired to reduce her opioid therapy. This case will describe the initiation of cannabis formulation containing 6 mg CBD and 6 mg THC per dose to illustrate how its use achieved a reduction in the patient’s overall pain while eliminating her opioid use.

## Case presentation

**Figure 1:**
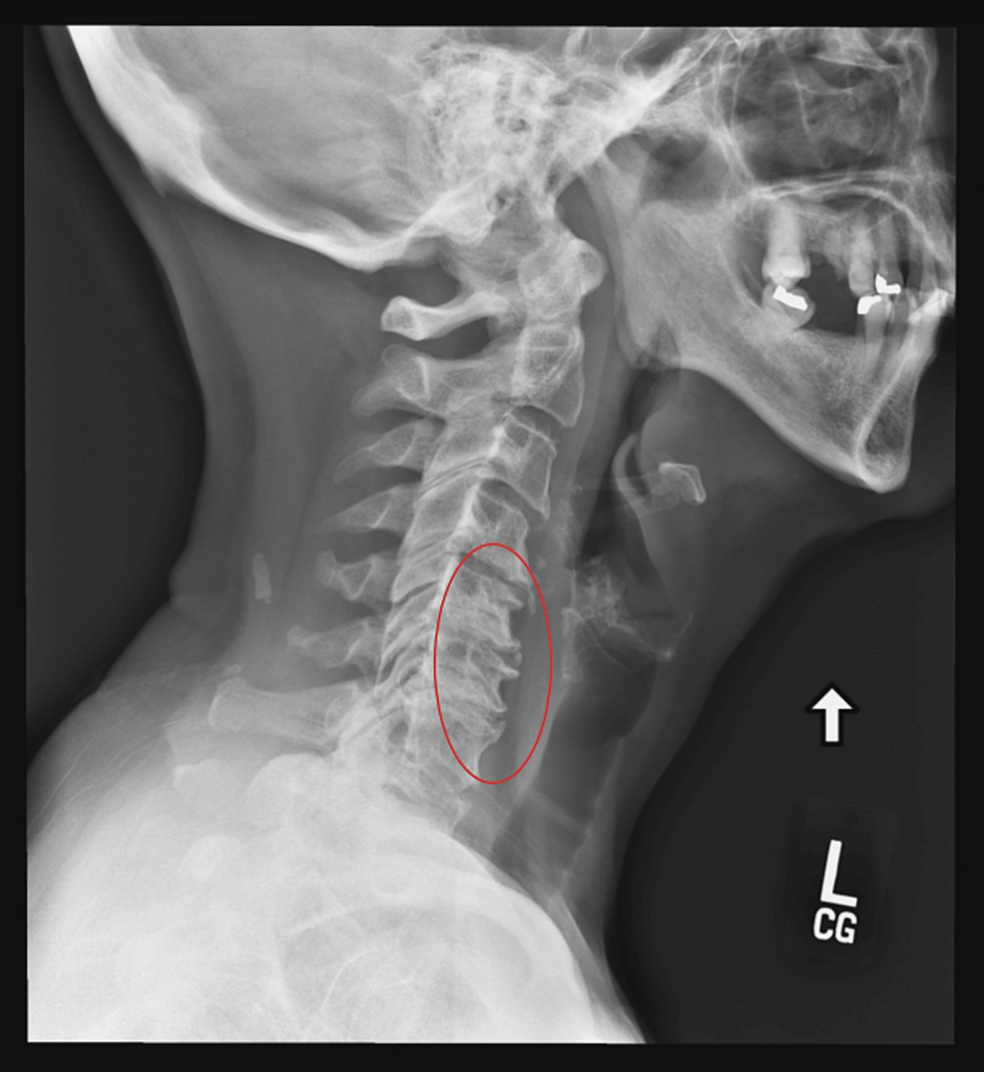
Lateral view X-ray of the cervical spine illustrating cervical spondylosis/degenerative changes (circled).

A 72-year-old female presented to her primary care physician with chronic neck and lower back pain and diffuse arthritic pain, secondary to scleroderma and osteoarthritis (OA). She had an extensive past medical history, including hypothyroidism, primary hypertension, hyperlipidemia, migraine, depression (recurrent without suicidality), insomnia, and osteopenia. Prior management with multiple oral medications, such as oxycodone-acetaminophen (10-325 mg) twice daily, oxycodone (20 mg, extended release 12 hours) twice daily, gabapentin (300 mg) up to five times daily, and intermittent cannabidiol ointment (dose not reported), ineffectively treated her pain. She stated that her lower back pain started around the age of 25, with no specific factor causing the onset indicated in her medical record. The patient at the time was healthy and did not have obesity. At times, the severity of the pain was forcing her to crawl around her house or at work due to exacerbations caused by standing and walking. The patient recently reported that her pain had markedly worsened, primarily in her lower back, and her neck pain became more severe and constant. Her BMI was 33.8 kg/m², and her examination was positive for diffuse tenderness in the lumbar area with associated paravertebral spasm. A cervical spine X-ray showed severe interspace narrowing at cervical spine vertebra C5/6 and C6/7 and moderate narrowing at C4/5 and overall cervical spondylosis/degenerative changes of the cervical spine (Figure 1).

The patient’s pain has been resistant to multiple treatment plans, resulting in increased opioid use, contributing to dependence and tolerance, which contributed to possible opioid-induced hyperalgesia. For 10 years, she was treated with oxycodone-acetaminophen (5-325 mg) four times daily and oxycodone (20 mg, extended release 12 hours) twice daily, which totaled approximately 90 morphine milligram equivalents (MME) daily. The patient repeatedly shared that she was determined to “not get hooked” on opioids. Within the last few years, the patient’s opioid regimen was changed to oxycodone-acetaminophen (10-325 mg) five times daily or 75 MME daily. Opioids moderately managed her pain, but she was worried about her chronic opioid usage. The patient was determined to reduce her opioid use.

In March of 2021, the patient was certified for medical cannabis initiation (due to chronic pain) as a cannabis-naïve patient in her primary care office. At the time of medical cannabis certification, she was utilizing two oxycodone-acetaminophen (10-325 mg) or 30 MME daily in an attempt to wean down to one tablet per day and a goal to eliminate opioid therapy. Her pain became a constant barrier to her quality of life, with increased severity associated with activities of daily living. During the certification process, her physician discussed various approaches to cannabinoid therapy, recommending slow up-titration of balanced 1:1 CBD/THC sublingual tincture. The patient was educated by the physician and given an initial symptom assessment scale to complete. In order to quantify the pain and quantity of improvement, an intake assessment was given to track the patient’s pain level. Pain level was measured on a scale from 1 to 10 and measured various aspects that may affect pain level. The system assessment scale measured level of pain, fatigue, nausea, depression, anxiety, drowsiness, appetite, well-being, shortness of breath, abdominal discomfort/bloating, and ability to move normally. For the purpose of this case report, we focused on reporting the patient’s pain level. At the patient’s initial interaction, she rated her pain level to be an 8 out of 10. The patient was then started on the 1:1 CBD/THC medical cannabinoid. A pharmacist at the dispensary confirmed the formulation and dose approach of the certifying physician and recommended cannabis products. The patient purchased a sublingual 1:1 tincture formulation following her consultation with the pharmacist at the dispensary.

The initial dosage was a sublingual balanced 1:1 CBD/THC tincture at 3 mg CBD and 3 mg THC three times daily. In the weeks to follow, the patient was contacted via phone to ensure the patient’s progress was recorded. The patient noted that she had no side effects to report and that her neck and back pain were “somewhat better.” Ultimately, this led to the patient’s increased dosage.

After one month, the patient increased the dose to 6 mg CBD and 6 mg THC sublingual formulation, three times daily. The patient stated that her pain became well controlled by the increased dose. At the patient’s last follow-up visit, she reported that her pain level scored a 6 out of 10. The patient stated that she was comfortably utilizing cannabis medicine in her home and took a dose before bed to improve pain and quality of sleep. She was regularly able to delay dosages if driving while still maintaining adequate pain relief. This formulation provided benefit for months with good efficacy, defined simply as a decreased level of pain compared to her initial visit. The patient then elected to purchase an “extra strength tincture” with increased THC concentration four times her usual dose, calculating to 12 mg THC, with her physician’s approval. After using this new higher-concentration formulation with the same three times daily frequency, she reportedly became dizzy and adamantly stated to her provider that the psychoactive effects were not what she wanted. She emphasized that she did not want to feel “high” or intoxicated by her cannabis. Following that experience, the patient decreased the volume of tincture she used during the day to an amount consistent with her efficacious dose to mitigate unwanted side effects due to the increased THC dosage.

The patient regarded the whole approach to using medical cannabinoid as an “adventure.” She overcame personal stigma associated with physician-recommended cannabis therapy but did not discuss her use among her social networks for fear of a “bad reputation” or judgment. The patient felt a sense of community at her dispensary and connected with others who utilized physician-guided cannabinoid therapy. In her experience, the more concentrated THC-only tincture formulation “tastes better without the CBD” and allows her to use less volume for palatability of dose.

Currently, this patient is doing well on cannabinoid therapy as recommended by her doctor. After nearly a year of medical cannabinoid use, she has not required additional opioids or NSAIDs but continues to utilize gabapentin (300 mg) up to five times daily as prescribed. According to our records, the patient’s oxycodone-acetaminophen prescription was last prescribed without refills 18 months ago and historically has been opioid-free with no further prescriptions evident in the system.

## Discussion

Medical cannabinoid formulation titration can prove difficult for patients as there lack specific guidelines in terms of dosing and formulation with medical cannabis certifications from physicians [4]. In addition, patients seeking an approach to medical cannabinoid therapy encounter multiple preparations of not only cannabis-based products, whole plant-based or purified tinctures, concentrates, pills, salves, or lozenges but also cannabis flower, which may be combusted or vaporized with variation in percentages of major/minor cannabinoids in addition to other pharmacologically active chemicals such as terpenes/terpenoids [12,13]. These limitations are likely influenced by the fact that medical cannabis is still federally illegal, and therefore, research is limited. Most patients benefit from a patient-centered conversation with their certifying physician in terms of expectations of chronic pain management and efficacy [4].

The patient in this case also suffers from systemic sclerosis, a progressive autoimmune disease with a presentation of fibrosis, extracellular matrix deposition, and vasculopathy. CB1 and CB2 receptors appear to have an influence on the modulation of disease; skin biopsies from humans show the overexpression of CB1 and CB2 receptors, with the activation of CB1 receptors appearing to exacerbate fibrosis [14]. CB2 activation may possibly provide protection against associated dermal thickening, inflammatory infiltration, and collagen accumulation [8]. Preclinical research shows that CB2 agonist therapy demonstrates the reduction of fibroblast and collagen accumulation, as well as decreased levels of inflammatory markers including transforming growth factor-beta 1 (TGF-β1), interleukin 6 (IL-6), tumor necrosis factor (TNF), and vascular endothelial growth factor (VEGF) [14].

Cannabinoid therapy is an approach sought by many older individuals suffering from chronic pain for conditions associated with osteoarthritis and degeneration. Finding non-opioid alternatives to treat chronic pain is a principle of philosophy for many clinicians [15]. Interestingly, the endocannabinoid system (ECS) appears to repair dysfunction in osteoarthritis, as synovial tissues express CB1 and CB2 receptors; patients with OA also have detectable levels of endogenous endocannabinoids, anandamide (AEA), and 2-arachidonoylglycerol (2-AG), in synovial fluid in comparison to samples from healthy individuals that do not [16]. Patients may find efficacy in utilizing cannabinoid therapy for the treatment of complex pain syndromes.

The findings of this case are limited since the data is based on the results of one individual. Cannabis plants and medical cannabis formulations vary immensely in terms of whole plant flower/extracts to chemical extractions and purified formulations. Additional chemical constituents (over 500 varied by plant chemotype) not recognized in this manuscript include minor cannabinoids, terpenoids/terpenes, flavonoids, phenolics, and alkaloids that may contribute to therapeutic effect. The concentration of cannabis-based formulations differs in terms of major and minor cannabinoids, and patients may utilize other routes of formulation, such as combustion or vaporization of flower, causing difficulty in determining the dose [12,13]. Although these results support the efficacy and safety of medical cannabis therapy as an alternative to opioid management, more studies are needed to determine the appropriate dosage, timing, and method of administration. Recent systematic reviews investigating the safety and efficacy of medical cannabinoid therapy for chronic pain have found that the certainty of evidence is low [17,18]. Of these reviews, one study stated that 20.8% of the patients reported a reduction in half or more of their pain and 3% of patients reported serious adverse events [17]. In the study, serious adverse events were defined as an event leading to hospitalization or death [17]. The other review found that chronic cannabinoid use, occurring for 24 weeks or longer, was associated with a higher rate of adverse events, which were defined as including nausea, vomiting, headache, drowsiness, and dizziness [18]. Serious adverse events including dependance, psychosis, and withdrawal symptoms were rare; a less serious but still notable adverse event, cannabis hyperemesis, is acknowledged [18]. These systematic reviews support the need for high-quality evidence and studies, including randomized control studies, to determine medical cannabinoid’s safety, efficacy, and dosage.

The importance of this case is that it demonstrates that medical cannabinoid therapy may be a potential opioid alternative for chronic pain management. Recently, opioid overdose deaths have significantly increased, totaling over 100,000 within the last two years [19]. The SARS-CoV-2 pandemic served as a fuel for this increased opioid-related mortality [20]. However, with alternative treatment modalities such as medical cannabis, there is an opportunity to combat this epidemic. Interestingly, one study looked at human immunodeficiency virus (HIV) patients with chronic pain and found that cannabis users had significantly lower odds of prescribed opioid use [21], suggesting that potential alternatives may reduce the usage of opioids. While this case study does not definitively indicate that medical cannabis is the best option for chronic pain management, it demonstrates that non-opioid alternatives can be a viable option to chronic opioid use. Future research is needed to determine their efficacy and safety profiles as alternative treatment options.

## Conclusions

This case indicates that cannabinoid therapy may be useful in managing chronic pain and therein reducing its detrimental impact on patients’ overall quality of life. Potential opioid alternatives, such as medical cannabis, are becoming increasingly important in pain management to improve patients’ quality of life. Additionally, this case demonstrates that medical cannabis may prove beneficial in reducing reliance on chronic opioid therapy. Clinicians should be aware of different approaches to treatment that do not include opioids.
